# Significance of Vibration Time in Developing Properties of Precast Pervious Concrete

**DOI:** 10.3390/ma16186239

**Published:** 2023-09-15

**Authors:** Karol Chilmon, Beata Jaworska, Maciej Kalinowski, Wioletta Jackiewicz-Rek, Aleksandra Podkoń

**Affiliations:** Faculty of Civil Engineering, Warsaw University of Technology, 00-637 Warsaw, Poland; beata.jaworska@pw.edu.pl (B.J.); maciej.kalinowski@pw.edu.pl (M.K.); wioletta.rek@pw.edu.pl (W.J.-R.); ola.podkon@gmail.com (A.P.)

**Keywords:** pervious concrete, compaction, porosity, water permeability, mechanical strength, vebe, consistency

## Abstract

Due to its properties, pervious concrete is usually considered a material of choice for permeable surfaces. However, its permeability properties, as well as mechanical performance, depend on its effective porosity. In this paper, the Authors investigated the influence of material and technological factors on the selected properties of pervious concrete. A new method, based on the Vebe consistency test method, was developed to assess the vibration time required to reach a designed effective porosity of pervious concrete. Five classes of pervious concrete’s consistency measured by the modified vebe method were proposed, and the limiting values to determine optimum vibration time were indicated. A model of dependence between the porosity of pervious concrete, its consistency, and compaction time was proposed. It was found that for the assumed range of variability, compaction time and material composition significantly influence the porosity of pervious concrete, and, therefore, all properties of pervious concrete.

## 1. Introduction

According to the “World Population Prospects 2019” report by the United Nations [1], 55% of the world’s population lives in cities. Forecasts from the same report indicate that the percentage of people living in cities will gradually increase in the coming years, and it is estimated that in 2050, about 75% of the entire population will live in cities. 

**Figure 1:**
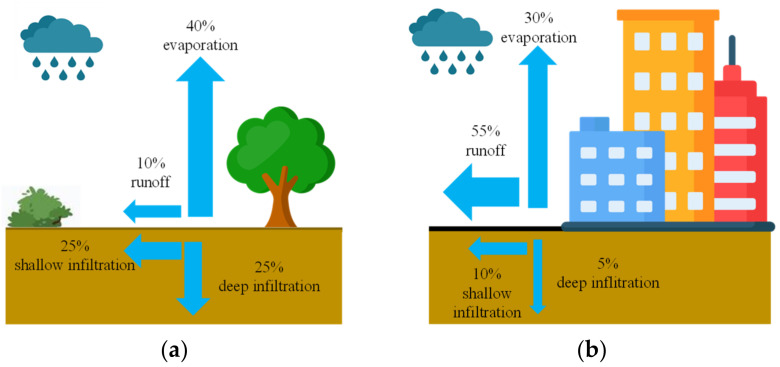
Water circulation in (**a**) natural environment and (**b**) urban environment with predominantly impervious pavement (95–100%).

Urbanization results in a successive reduction of biologically active areas, which are usually replaced by non-permeable surfaces. About 55% of rainwater from non-permeable surfaces is estimated to be discharged to sewage systems, and the other 30% evaporates. Consequently, only about 15% of the total rainfall soaks into the ground [2]. For comparison, about 50% of rainwater soaks into the ground in natural conditions (Figure 1).

Replacing natural vegetation areas with impervious land covers, such as conventional concrete and asphalt surfaces, contributes to water shortages during meteorological droughts and floodings during storm-type rains. The lack of water and air permeability of conventional pavement also disturbs the exchange of heat and moisture between soil and air, which causes the phenomenon of hot islands in urban areas and contributes to the deterioration of human health [3].

Permeable pavements (PP) made of such materials as pervious concrete, porous asphalt, unit pavers, or granular materials are essential to reduce stormwater runoff, recharge groundwater supplies, and reduce the impact of the urban heat islands [4]. Unlike regular concrete modified in various ways [5], an open porous structure makes pervious concrete a particular type that allows water to flow freely through its structure. Pervious concrete has been successfully used for years in road and pedestrian water-permeable solutions in the U.S., Japan, Canada, and many European countries [6]—pervious concrete pavements allow quick and effective drainage of rainwater to the ground or an additional rainwater recovery system and enable cleaning and cooling of water during its flow through successive pavement layers. According to research [7], a square meter of porous concrete pavement can drain from 80 to 730 L of water per minute, which allows for a significant reduce the phenomenon of surface runoff, even in the case of heavy rainfall, while being able to remove up to 90% of total suspended solids [8] and heavy metals [9] from stormwater runoff, making it safe for discharge into water reservoirs or the surrounding ground without the need of further treatment.

Pervious concrete pavements can be cast in place or made from precast elements. Precasting allows better quality of pervious concrete elements due to the controlled environment in which they are made and the restricted quality control, which is often impossible when casting concrete in situ [10]. Pervious concrete pavement made of precast elements is also easier to repair since precast slabs allow removal and replacement as part of routine maintenance. However, several technological issues must be addressed to ensure the proper performance of pervious concrete, the compaction method being one of them.

**Figure 2:**
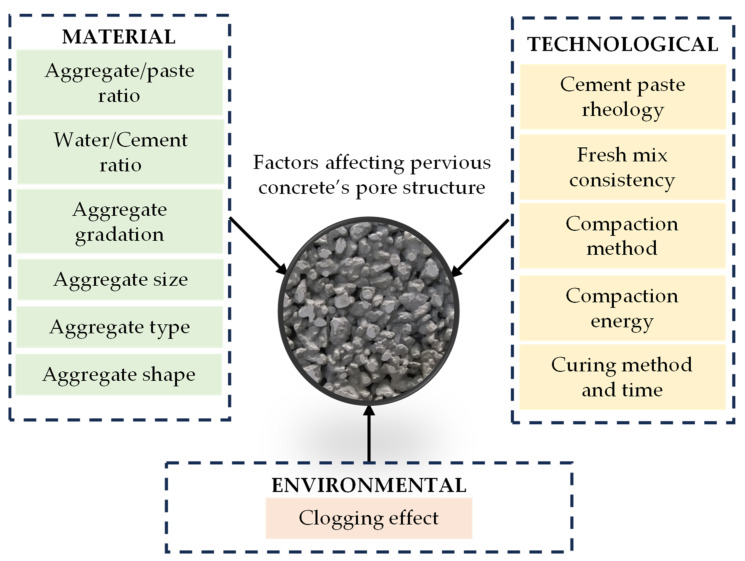
Factors affecting pervious concrete’s pore structure.

The pore structure is the crucial parameter affecting pervious concrete’s mechanical, hydrological, acoustic, and durability performance [11,12]. The pervious concrete pore structure may be characterized by different factors, such as pore volume, pore size, connectivity, and tortuosity [13]. However, due to ease of measurement, the most commonly used characteristics are those regarding pore volume, such as effective (or open) and total (or overall) porosity [14]. Total porosity is usually calculated based on the fresh mix density, and the effective porosity is measured on hardened samples using f.e. hydrostatic methods [15]. Controlling those characteristics is crucial in obtaining the desired and stable properties of precast or in situ pervious pavements. Studies have shown [16,17,18,19,20,21] that pervious concrete’s pore structure depends on various factors (Figure 2), which could be categorized into material, technological, and environmental.

Except for material factors describing the composition of pervious concrete, which contribute significantly to its performance, the applied compaction method and energy are critical in influencing the pore structure of pervious concrete [18,21] and its overall properties. Compared to regular concrete, pervious concrete is characterized by a comparatively large amount of interconnected air pores (up to 30% of material volume), allowing for rapid water flow through the concrete’s structure—due to this, the amount of regular concrete’s components (mainly aggregate) in the case of pervious concrete per 1 m^3^ is much lower.

In situ pervious concrete is compacted mainly using hand steel rollers with low compaction energy directly after pouring [22]. This type of application is limited to bikeways, parking lots, and roads with low traffic [23], as low compaction energy results in a thin layer of cement paste covering the aggregates and often insufficient packing of aggregates. Increasing the compaction energy leads to close packing of aggregates and a thicker cement paste layer around the aggregates [24]. This results in lower porosity and significantly improves pervious concrete’s mechanical properties and durability [25]. Bonicelli et al. [26] suggested the usage of drum roller compaction to lower the porosity of the in situ pervious concrete. However, as the Authors pointed out, timing, weight of the roller, and number of passes should be correctly assessed as using too much energy may destroy the structure of hardening concrete. Precasting pervious concrete allows one to choose from various compaction methods, such as vibrating tables or concrete block machines. However, using those techniques requires carefully assessing the vibration frequency, amplitude, and time.

Unlike conventional concrete, choosing the optimal parameters for the vibration process of pervious concrete is much more difficult since those are highly dependable on fresh mix workability. Vibrating fresh mix with high consistency for even a short time often leads to cement paste sagging and clogging the bottom of the sample, while mixes with lower consistency can be vibrated longer with no such risk. Compacting process parameters are often chosen via trial and error as there is a lack of standard test methods for measuring pervious concrete’s workability characteristics.

Several articles regarding the pervious concrete compaction using the vibration method have been published—Chindaprasirt et al. [27] reported a significant decrease in void ratio with increasing vibrating energy. The void ratio decreased from 50% to slightly lower than 35% and 30% for vibrating energy of 6 and 36 kNm/m^2^. Increasing vibrating energy further to 90 kNm/m^2^ resulted in a void ratio equal to 25% and compressive strength in the 15–22 MPa range, depending on the paste flow values. In their studies, Chindaprasirt et al. [27] applied the surface vibration method used in concrete block machines. The authors suggested 10 s with a vibrating energy of 90 kNm/m^2^ as optimal in compacting pervious concrete. Yuan Jie Zhu et al. [25] investigated the properties of pervious concrete samples molded in two layers, each vibrated for 3, 5 and 7 s, respectively, for a total of 6, 10, and 14 s. Authors reported a decrease in sample porosity from 20 to 7.8% when increasing the total time of vibration from 6 to 14 s, which resulted in higher compressive strength (from 16.7 to 35.8 MPa) but significantly lower water permeability (water permeability coefficient from 5.7 to 0.9 mm/s). Such low water permeability indicated that samples that vibrated for 14 s were clogged at the bottom. Yuan Jie Zhu et al. [25] suggested vibration time between 8 and 12 s as optimal for achieving the desired properties of pervious concrete. Unfortunately, in neither of the articles mentioned above, the frequency and amplitude of the vibrating table were reported.

Suleiman et al. [28] used a two-stage technique of compaction. Before 5 s vibration on the vibration table, samples were compacted by rodding 25 times in three layers. The authors applied two different vibration amplitudes of 0.127 and 0.086 mm to differentiate the compaction energy. An increase in vibration amplitude resulted in an 11–31% lower void ratio, depending on the initial porosity. Samples with lower porosity have a 28–54% higher compressive strength and significantly better freeze and thaw resistance. Samples compacted with higher energy failed after 153–196 freeze and thaw cycles (acc. to the ASTM C666–procedure A [29]), while samples compacted with lower energy failed after 110 cycles.

Pervious concrete technology presents a different, environmentally friendly aspect of concrete technology [30]. Usually, when designing concrete elements, the focus is put on its mechanical performance, durability, and carbon footprint—typically, no other functions of the component are designed or thought about. With pervious concrete, especially when used as a pavement material, its functionality can be extended to different aspects of water management (water drainage, purification, and/or storage during droughts). However, to guarantee the designed performance of pervious concrete, its preparation process must consider the dependence between its properties, porosity, and technological compaction process. The presented study aims to investigate the significance of vibrating time on the developing properties of pervious concrete. The authors studied the influence of different cement paste content, fresh mix consistency, and vibration time on pervious concrete’s porosity and mechanical properties. A novel approach based on the vebe method [31] was proposed to measure the pervious concrete consistency and determine optimum compaction time considering compacted concrete mix properties.

## 2. Experimental Plan and Methods

### 2.1. Experimental Plan

**Figure 3:**
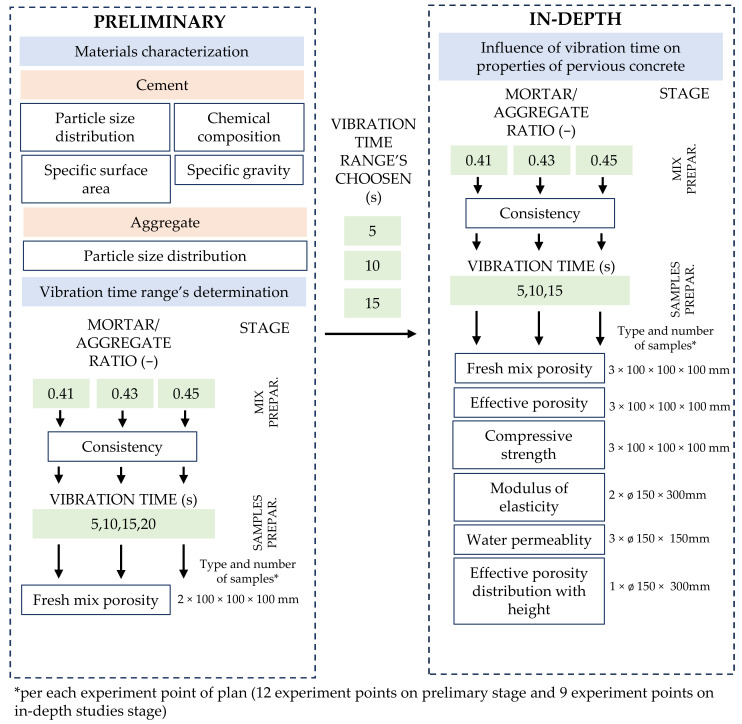
Experimental framework with values of dependent variables, list of conducted tests, and samples used.

The experimental plan consisted of two stages: preliminary and in-depth studies (Figure 3). The preliminary research aimed to determine the characteristics of raw materials and the range of vibration’s time. Two full factorial experiments were designed, each with two independent variables: mortar-to-aggregate mass ratio and vibration time. Three values of mortar (cement + sand + water) to aggregate ratios were adopted—0.41, 0.43, and 0.45. Prepared mixes were compacted via vibration method for different times—5, 10, 15, and 20 s, respectively. Based on the Authors’ findings, the vibration time range for the in-depth phase was narrowed to 5, 10, and 15 s. Dependent variables in the preliminary phase were consistency (acc. to the modified vebe method) and the porosity of fresh mix (acc. to a gravimetric method). The in-depth studies stage consisted of 6 dependent variables—consistency (acc. to modified vebe method), porosity of fresh mix (acc. to gravimetric method), effective porosity (acc. to hydrometric method), compressive strength (acc. to PN-EN 12390-3 [32]), modulus of elasticity (acc. to ASTM C469 [33]), water permeability (acc. to falling head method) and effective porosity distribution with height (acc. to hydrometric method).

The following constants were adopted at both stages of the research: water-to-cement ratio (mass), cement-to-sand ratio (mass), method of mixes and samples preparation, and methods of sample curing and testing.

Three types of samples were used in the research: 100 × 100 × 100 mm cubic samples and ø150 × 300, ø150 × 150 mm cylindrical samples. The amount of samples in each of the tests varied from 1 to 3.

### 2.2. Methods

#### 2.2.1. Raw Material Characteristics

Cement’s particle size distribution was measured by a laser diffraction method on the particle size analyzer LA-300 (Horiba, Tokyo, Japan). A total of 0.4% P_2_O_5_ solution was used as a dispersant, and 60 s of ultrasounds were applied to the cement’s dispersion before testing. The chemical composition analysis of cement was performed by X-ray fluorescence method using an Epsilon3x spectrometer (Malvern Panalytical Ltd, Malvern, UK). Cement’s specific surface was measured using Blaine’s method, and its specific gravity was determined by the pycnometer’s method. The sieving method determined the sand’s particle size distribution according to PN-EN 933-1 [34].

#### 2.2.2. Concrete Samples Preparation and Curing

Samples were formed in one layer and compacted on a vibration table for a predefined period. The vibration frequency was 50 Hz, and the average vibration amplitude was 1.0 +/− 0.2 mm. During the compaction process, molds were fixed to the table. Before demolding, samples were stored for 24 h in the curing chamber (Temp = 22 ± 2 °C, RH > 95%). After demolding and prior to tests, samples were cured for 28 days in a curing chamber (temp = 22 ± 2 °C, RH > 95%).

#### 2.2.3. Consistency

**Figure 4:**
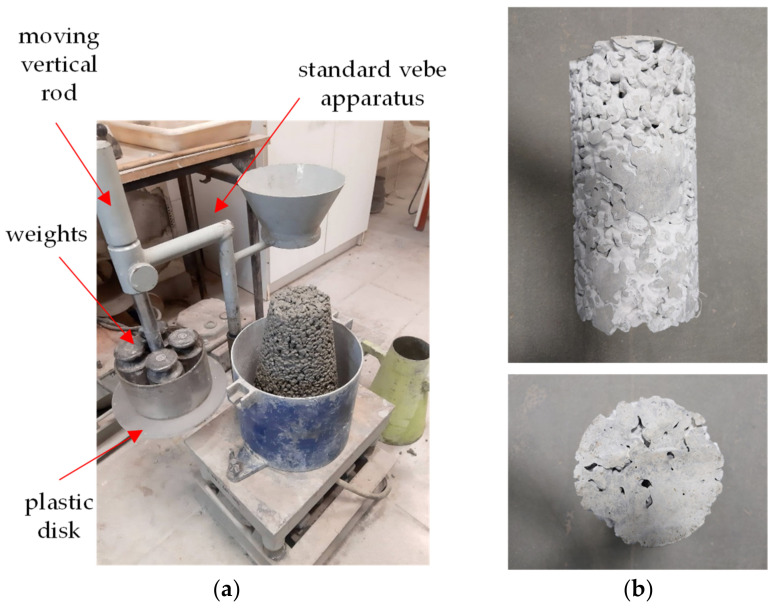
(**a**) Modified vebe apparatus used in the research and (**b**) clogging effect due to too long vibration time.

The consistency of fresh mix was tested using a modified vebe method, similar to the ASTM C1170-06 [35] test method used to control the consistency of roller-compacted concrete. The standard vebe apparatus (50 Hz frequency and 0.5 +/− 0.1 mm amplitude) was modified so that the plastic plate was uniformly loaded with a weight of 8.0 kg (Figure 4a). The overall load (additional weights, plastic disk, and moving vertical rod) transferred to the sample was approx. 12.0 kg, which corresponded with the approximate mass of the tested pervious concrete sample. The method aimed to determine the time needed to cement paste sagging and clogging effects occur (Figure 4b).

The modification allowed to replicate in near-to-surface layers similar compaction conditions to those occurring typically at the bottom of the mold where the wall effect and cement paste sagging clogging effect takes place. The test consisted of a few steps:

1. Form the slump’s cone into three layers, each compacted by rodding 25 times;
2. Raise the slump’s cone and spread the fresh mix evenly in the vebe container;
3. Adjust the weighted plastic disc on the top of the fresh mix and start vibration;
4. Measure the time until the cement paste fills the bottom of the transparent plastic disk or starts to sag downwards visibly.

#### 2.2.4. Fresh Mix Porosity

Fresh mix porosity P (%) was calculated based on the difference between the measured pervious concrete’s density and the theoretical density of concrete mix with assumed 0% porosity by Equation (1).

(1) $$P=\left(1-\frac{\rho_{m}}{\rho_{t}}\right)\cdot 100\%,$$

where:

$\rho_{m}$—fresh mix measured density, kg/dm^3^.

$\rho_{t}=\frac{C+W+FA+CA}{\frac{C}{\rho_{C}}+\frac{W}{\rho_{W}}+\frac{FA}{\rho_{FA}}+\frac{CA}{\rho_{CA}}}$—theoretical density of mix with 0% porosity, kg/dm^3^.

C,W,FA,CA—cement, water, fine aggregate, and coarse aggregate masses, respectively, kg.

$\rho_{C},\;\rho_{w},\rho_{FA},\;\rho_{CA}$—cement, water, fine aggregate, and coarse aggregate specific gravities, respectively, kg/dm^3^.

The fresh mix’s density was measured after compaction in 100 × 100 × 100 steel forms.

#### 2.2.5. Effective Porosity and Effective Porosity Distribution with Height

**Figure 5:**
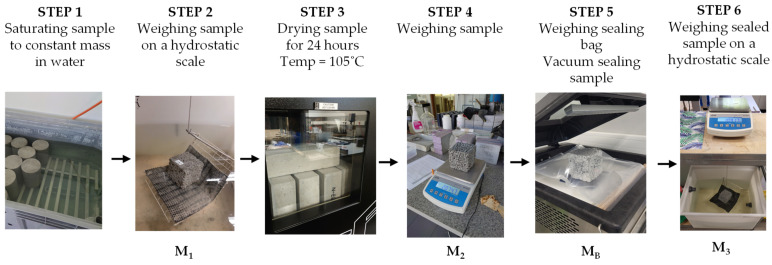
Effective porosity determination flow chart.

Effective porosity was measured using the hydrostatic method. The technique consisted of several steps (Figure 5).

First, samples were saturated to constant mass in water, and then the hydrostatic mass of the samples was measured. Afterward, samples were dried at a constant temperature of 105 °C for 24 h (when developing the procedure, 24 h of drying was found sufficient to dry pervious concrete effectively). Next, the samples’ dry mass was measured. Then, the sample was placed in a plastic bag and sealed in a vacuum using the Corelok device (InstroTek Inc., Raleigh, NC, USA). A dry sample prepared that way was then placed in a hydrostatic balance. The collected results were used to calculate the porosity of the samples. The effective porosity PE (%) was calculated according to Equations (2)–(4).

(2) $$P_{E}=\frac{V_{sb}-V_{s}}{V_{sb}}\cdot 100\%,$$

where

$V_{sb}$—the total volume of the sample (open pores and material’s skeleton), cm^3^.

$V_{s}$—material’s skeleton volume, cm^3^.

(3) $$V_{S}=\frac{M_{1}-M_{2}}{\rho_{W}},$$

where

$M_{1}$—the mass of the saturated sample on the hydrostatic scale, g.

$M_{2}$—mass of dried sample, g.

$\rho_{W}$—water’s specific gravity, g/cm^3^.

(4) $$V_{SB}=\frac{M_{2}+M_{B}-M_{3}}{\rho_{W}}-V_{B},$$

where

$M_{2}$—mass of dried sample, g.

$M_{B}$—mass of sealing bag, g.

$M_{3}$—the mass of the sealed sample on the hydrostatic scale, g.

$\rho_{W}$—water’s specific gravity, g/cm^3^.

$V_{B}$—sealing bag’s volume, cm^3^.

**Figure 6:**
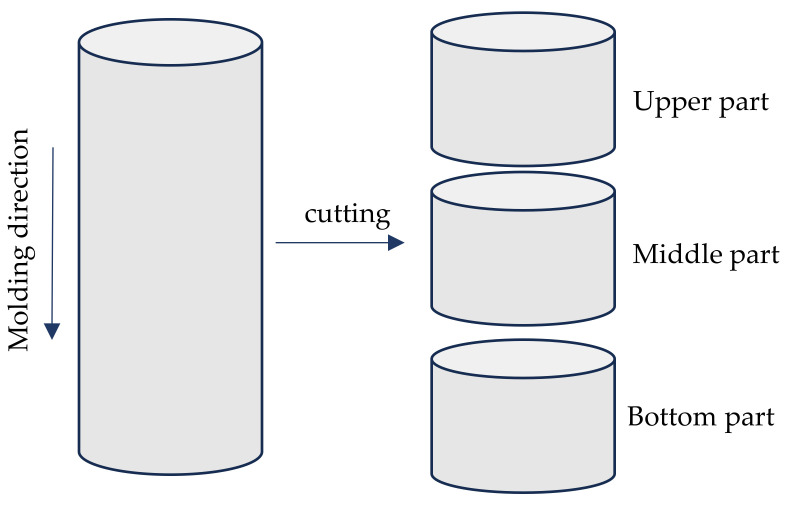
Sample preparation scheme for determining porosity’s distribution with height.

Cylindrical samples (ø150 × 300 mm) were used to determine the porosity’s distribution with a sample’s height. After 28 days of curing, samples were cut into three parts, each 95 mm long (Figure 6). The samples thus obtained were cleaned after cutting with a hard brush. The effective porosity of each part was determined using the 6-step procedure described above.

#### 2.2.6. Compressive Strength and the Modulus of Elasticity

**Figure 7:**
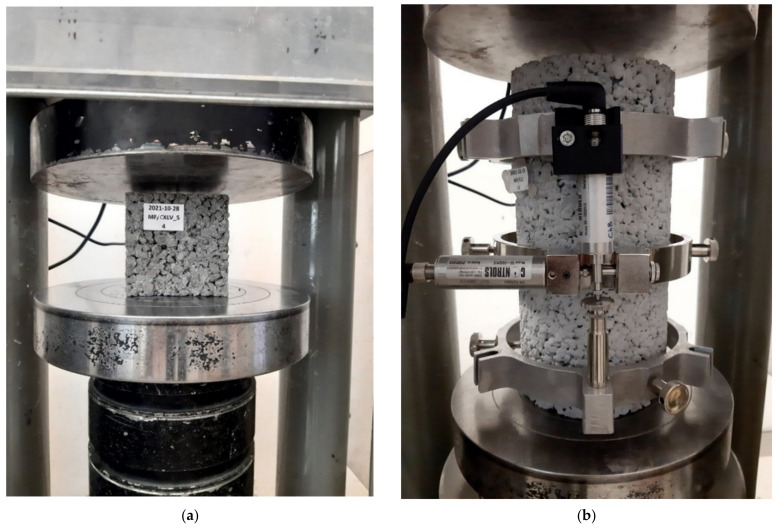
Pervious concrete sample during (**a**) compressive strength and (**b**) modulus of elasticity test.

Compressive strength was tested according to the PN-EN 12390-3 [32] method using the CONTROLS AUTOMAX (CONTROLS S.p.A, Milano, Italy) testing machine. The modulus of elasticity was tested according to ASTM C469 [33]. Cubic samples with 100 × 100 × 100 mm dimensions were used for compressive strength tests, and cylindrical samples with 150 mm diameter and 300 mm height were used for determining modulus of elasticity (Figure 7). A maximum of 40% of the ultimate load was applied to the sample in the test.

#### 2.2.7. Water Permeability

The falling water head method was used to measure the permeability of pervious concrete on cylindrical samples with a diameter of 150 mm, and height of 150 mm using a self-made falling head system. The water permeability was defined by filtration coefficient k (mm/s) and calculated according to Equation (5).

(5) $$k=\frac{a}{A}\cdot \frac{L}{t}\cdot \ln(\frac{h_{1}}{h_{2}}),$$

where

*a*—cross-sectional area of the cylindrical measuring tube, mm^2^.

*A*—cross-sectional area of a cylindrical sample (in calculations, the a/A coefficient was one due to the appropriate design of the measuring device), mm^2^.

*L*—cylindrical sample height, mm.

*t*—time of water column fall from h_1_ to h_2_, s.

*h*_1_—the initial height of the water column from which time measurement begins, mm.

*h*_2_—the final height of the water column at which measurement ends, mm.

**Figure 8:**
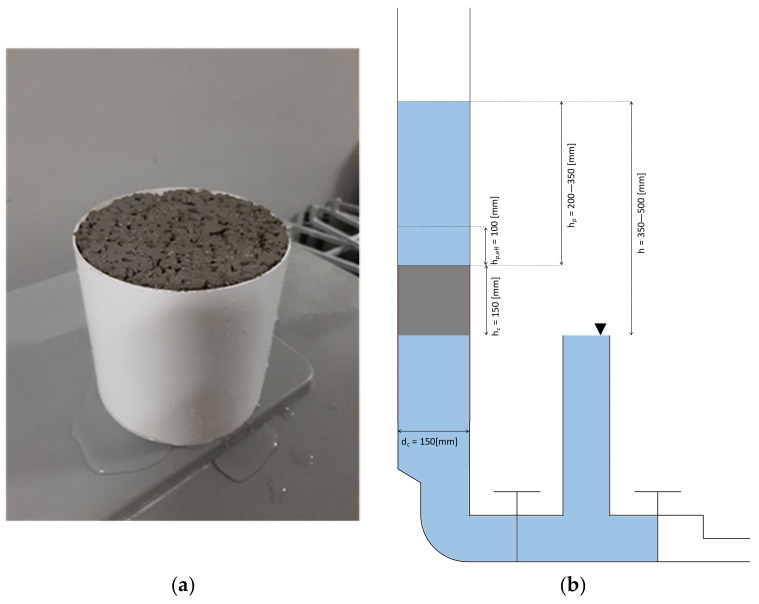
(**a**) Sealed pervious concrete sample after water permeability test and (**b**) scheme of apparatus used to determine pervious concrete’s water permeability.

The side surfaces of the samples were sealed during molding using plastic cylinders to eliminate the free water flow in the space between the sample and the test device (Figure 8a). Such a solution allowed a more accurate determination of the filtration coefficient. Due to the laminar flow assumption, the h_1_ height was located 100 mm from the sample’s upper surface, and the h_2_ height was located at the sample’s surface (Figure 8b).

## 3. Materials and Mixture Composition

### 3.1. Materials

#### 3.1.1. Cement

**Figure 9:**
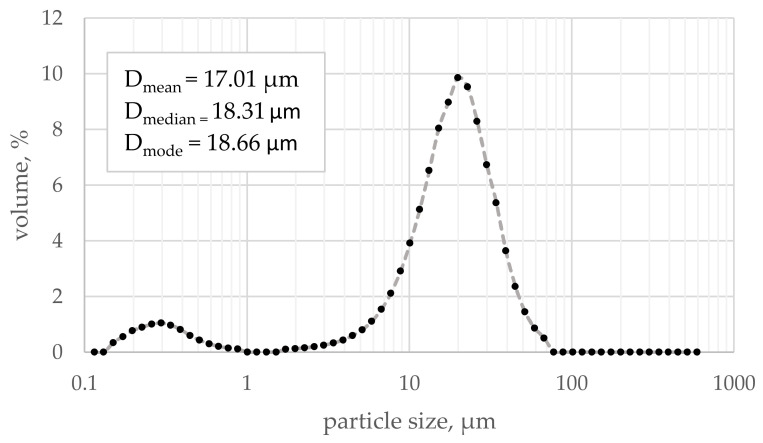
Particle size distribution of cement used in the research.

CEM II/A-S 52.5R compliant with PN-EN 197-1 [36] from a cement plant near Karsy town (Karsy town, Poland, Cement Ożarów S.A, CRH group) was used. According to Blaine’s method, the cement’s average specific surface area was 4740 cm^2^/g, and the average specific gravity was 3.06 g/cm^3^. The chemical composition is given in Table 1, and the particle size distribution is shown in Figure 9.

#### 3.1.2. Aggregate

**Figure 10:**
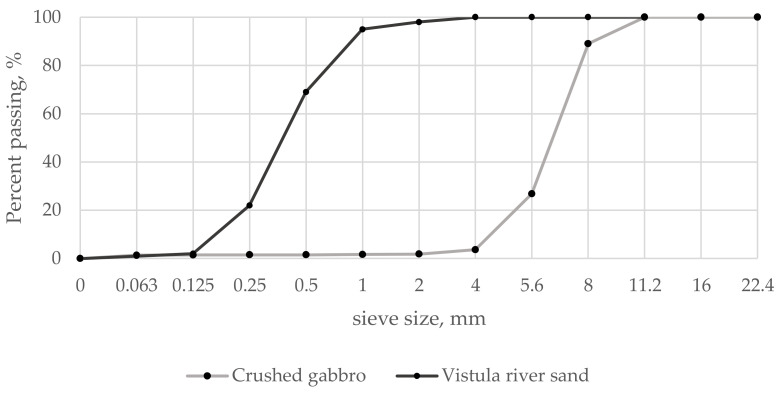
Sieving analysis graph of aggregates used in the research.

Two types of aggregates complaint with PN-EN 12620 [37] were used in the studies: Vistula River sand (0/2 mm fraction, Polbot company, Warszawa, Poland) and crushed gabbro (4/8 mm fraction, Nowa Ruda-Słupiec mine, Nowa Ruda, Poland). The particle size distributions of aggregates are shown in Figure 10.

#### 3.1.3. Water and Admixture

Tap water complaint with PN-EN 1008 [38] was used. PCE-based superplasticizer BASF ACE 430 complaint with PN-EN 934-2 [39] was used to improve the workability of the fresh mix.

### 3.2. Mixture Composition

Three mixtures were designed with a constant water-to-cement ratio of 0.30 and a constant cement-to-sand ratio of 1.618. The mixtures differed from each other in mortar to coarse aggregate ratio. Values of 0.41, 0.43, and 0.45 were adopted, respectively. The superplasticizer’s content was adjusted based on the observation of fresh mix cohesion in the handball rolling test [40]. Given the different mortar-to-aggregate ratios, the amount of fine aggregate in each mix differed from 13.3% to 14.4% of the total aggregate. As the exact amount of each component per cubic meter depends on fresh mix porosity after compaction, only significant proportions defining each mixture are shown in Table 2. The laboratory recipes of the described mixes, calculated for cubic meters, are presented in the discussion section.

## 4. Results

### 4.1. Preliminary Research

#### 4.1.1. Consistency

It was assumed that the result of the consistency test for pervious concrete, unlike regular concrete, should determine the optimal technological process of its production and allow for the mitigation of phenomena deteriorating its properties—to examine the susceptibility of pervious to clogging during compaction. Consistency, as a relative ability of freshly mixed concrete to flow, was determined by the modified vebe method in which a specified volume of the fresh mix was subjected to vibration (50 Hz and 0.5 mm amplitude). Vebe time was defined as the time required for the transparent disc to be fully covered with cement paste or as the time required for cement paste to sag downwards visibly, whichever occurred first.

**Figure 11:**
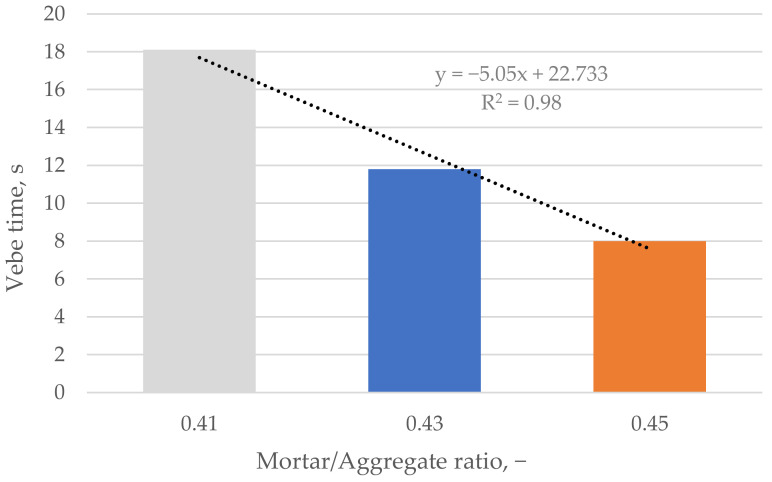
Vebe time vs. mortar/aggregate ratio. Colors indicate the mortar to aggregate ratio.

Vebe time varied from 8.0 to 18.0 s for prepared concrete mixes and decreased linearly with increased cement paste content. The longest time was obtained for the mixture with the lowest mortar-to-coarse aggregate ratio (Figure 11).

Vebe time of each mixture was within the range of quantification for this method, which suggested the assumed load was sufficient. After the modified vebe test, each sample showed significant signs of overcompaction—clogging.

#### 4.1.2. Fresh Mix’s Porosity

**Figure 12:**
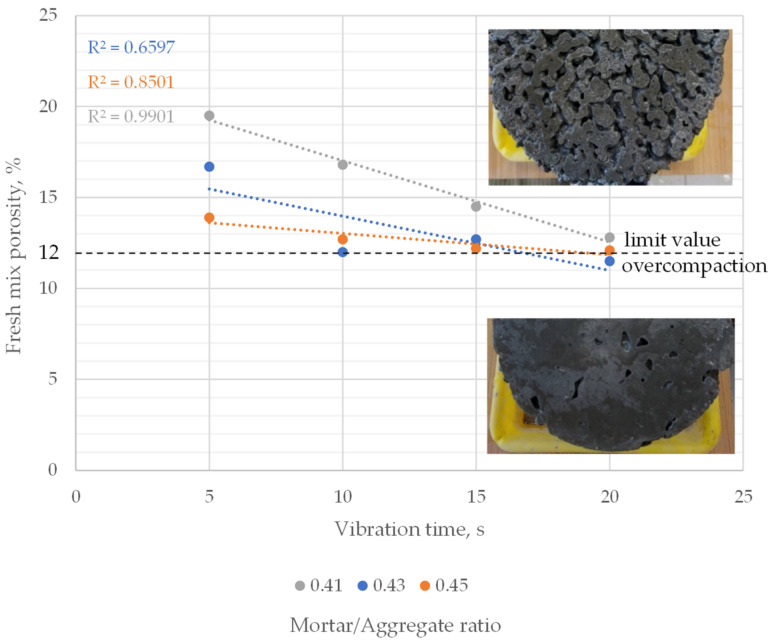
Fresh mix porosity vs. vibration time for mixes with varying mortar to aggregate ratio.

Fresh mixes’ porosity in the 11.5–19.5% range was observed. Porosity decreased linearly with an increase in vibration time. However, the significance of changes was different for each mix. The highest change in porosity was observed in the mix with the lowest mortar-to-aggregate ratio, where approximately a 12–13 percent decrease in porosity per 5 s of vibration was observed. Changes in the case of the two other mixes were significantly lower. Except for the outlier (porosity of 0.43 mortar to aggregate mix after 10 s of vibration), 1–9% decreases were noted per 5 s vibration (Figure 12).

The lowest obtained porosities for each mix were similar regardless of the mortar-to-aggregate ratio and were approximately 11.5–12.5%. Samples with such low porosities were visibly clogged at the bottom and sides. Hence, 12.0% fresh mix porosity was adopted as the limit value, indicating overcompaction of pervious concrete for further research.

Based on the detrimental effect of prolonged compaction on porosity characteristics of pervious concrete for the next research stage, the compaction time range was narrowed to a maximum of 15 s. For the assumed rheological properties of the cement mortar and superplasticizer content used in this study, it was assumed sufficient to study properties of pervious concrete, both in fresh and hardened state.

### 4.2. In-Depth Research

Based on preliminary research, it was found that for the assumed range of variability of mortar content in pervious concrete, its composition and fluidity, compaction by vibration exceeding 15 s resulted in clogging concrete’s structure. As such, it was deemed unnecessary to include such a long compaction time in the next phase of the experiment, as the acquired material would no longer be able to perform the functions of a pervious concrete.

#### 4.2.1. Consistency

Three batches of each mix were prepared during in-depth research, and the consistency of three samples from each batch was tested. Vebe time varied among mixtures, from 20.4 to 22.1 s in the case of the mix with the lowest mortar content, from 10.4 to 15.0 s with the 0.43 mortar to aggregate ratio, and from 5.1 to 7.8 s with the 0.45 mortar to aggregate ratio. Vebe time decreased linearly with the increase of mortar to aggregate ratio. For the mix with the highest amount of mortar, only 5.1–7.8 s was enough for the clogging effect to occur, unlike the mix with the least amount of mortar, which could be compacted for 20 s without the clogging effect.

**Figure 13:**
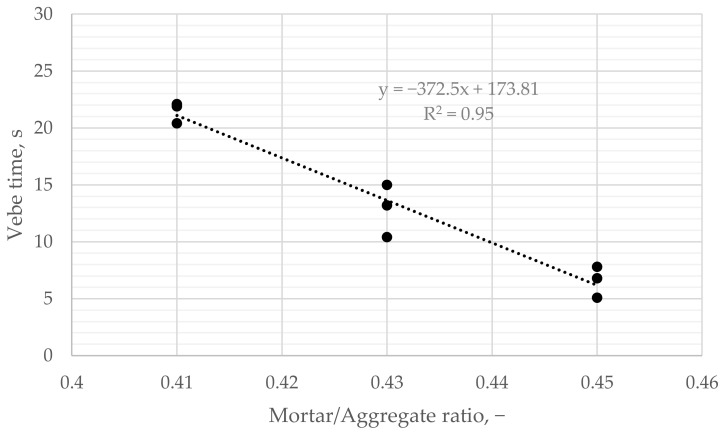
Vebe time vs. mortar/aggregate ratio.

It was observed that depending on the amount of mortar in the composition of the mix, the rheological behavior of the fresh mix changed, and the test via the proposed modified vebe method allowed us to quantify those differences (Figure 13).

#### 4.2.2. Fresh Mix and Effective Porosity

The porosity characteristic of pervious concrete determines its properties, both mechanical and regarding water permeability. In the case of performed research, fresh mix porosity and effective porosity of hardened concrete were measured. It was found that depending on the mortar content in prepared mixes, as well as vibration time, both the total fresh mix porosity and effective porosity significantly changed (Table 3). The Highest fresh mix porosity—18.44%—was observed for concrete mix with the lowest mortar-to-aggregate ratio—0.41, vibrated for 5 s. On the contrary, the lowest fresh mix porosity was observed for concrete mix with the highest mortar-to-aggregate ratio—0.45, vibrated for 15 s.

**Figure 14:**
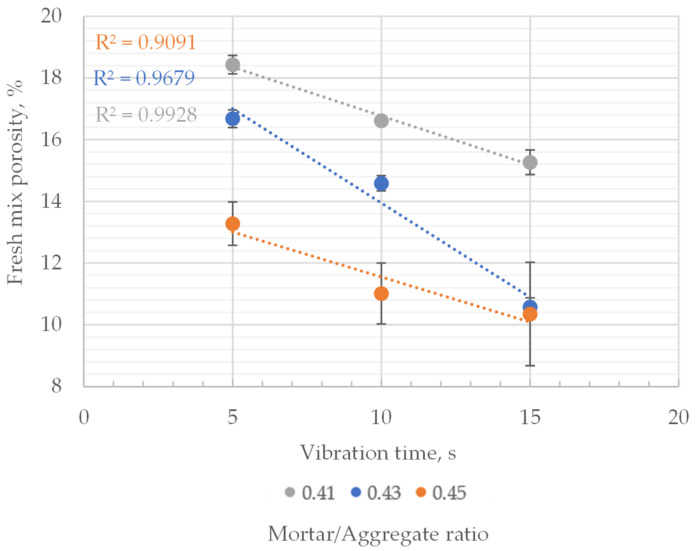
Influence of vibration time on the porosity of fresh concrete mix of different material compositions.

For each set of mixes characterized by the same mortar-to-aggregate ratio, a dependence between vibration time and change in porosity was observed (Figure 14). With an increase in mortar content within pervious concrete composition, the porosity of fresh mix decreased significantly, regardless of vibration time. The average porosity decreased with an increase of the mortar-to-aggregate ratio of the composite: from 18.44% to 13.27%, in case of samples vibrated for 5 s, from 16.68% to 10.57% in case of samples vibrated for 10 s and from 13.27% to 10.35% in case of samples vibrated for 15 s.

In the case of compaction time’s influence on the porosity of fresh mixes of the same mortar-to-aggregate ratio, it was linearly dependent on the vibration time. This case was valid for all analyzed concrete mixes. The most substantial effect of compaction on the porosity characteristics was observed for mixes characterized with a mortar-to-aggregate ratio of 0.43, where the porosity of fresh mix changed from 16.68% to 10.57% with an increase in vibration time (from 5 to 15 s).

**Figure 15:**
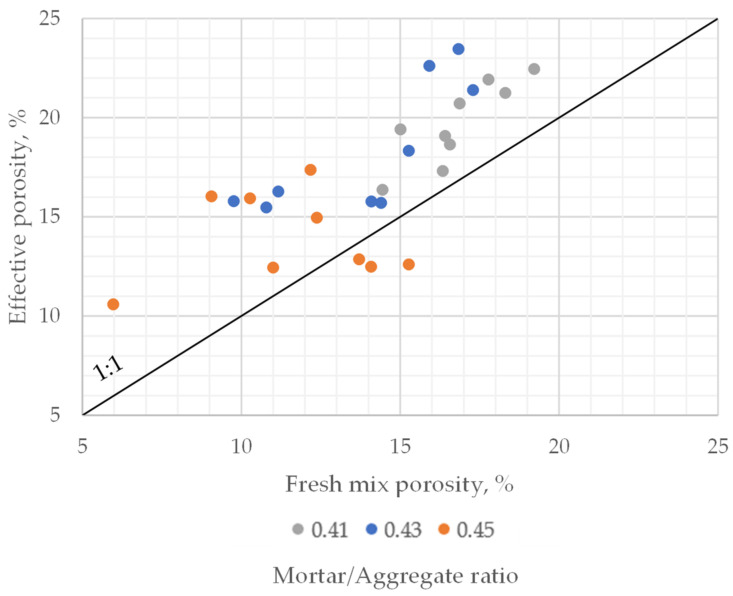
Fresh mix porosity vs. effective porosity of hardened pervious concrete. Each research point shows porosities of cementitious material in different states for 100 × 100 × 100 mm concrete samples.

For the same samples on which the porosity of fresh mix was tested, after 28 days, tests regarding the effective porosity of hardened pervious concrete were performed. This effort was made to establish a link between both those properties. It was found that the porosity of fresh mix was almost always lower than the effective porosity of hardened pervious concrete (Figure 15). This case was always valid for concrete series with a mortar-to-aggregate ratio of 0.41 and 0.43. For mixes characterized with a 0.45 ratio, which were mostly clogged, the effective porosity of hardened pervious concrete was lower than the porosity of fresh concrete mix in several cases.

#### 4.2.3. Effective Porosity Distribution

Cylindrical samples with a diameter of 150 mm and height of 300 mm were prepared to investigate the distribution of pores within different layers of compacted pervious concrete. After curing, samples were cut into three layers of the same height—upper, middle, and bottom (U, M, and B) (Table 4). Both compaction time and mortar-to-aggregate ratio had a statistically significant effect on the average values of porosity of hardened pervious concrete. It was found that the average effective porosity decreased with an increase in compaction time for concretes characterized by the same material composition. Also, the effective porosity decreased with an increase in the mortar-to-aggregate ratio of concrete for series that were compacted for the same time. On average, the effective porosity of cut layers from cylindrical samples was significantly higher than the corresponding effective porosity measured on 100 × 100 × 100 mm cubic samples.

In each tested series, the porosity varied between different layers of prepared samples. In most cases, the layer with the highest porosity was the bottom one—the one closest to the compaction table during sample preparation. The porosity of the upper part of the sample was the lowest one in all tested samples. The difference in effective porosity of different layers collected from the same sample was significant, reaching 8.57% (from 11.99% to 20.56% for different layers of a sample vibrated for 10 s and characterized by mortar-to-aggregate ratio of 0.45). Series with a mortar-to-aggregate ratio of 0.45 compacted for 15 s was characterized by the smallest difference in effective porosities between different layers. Although the entire sample was characterized with the lowest porosity of all tested samples (average of 11.72%), the difference in this property between layers reached only 3.58% (from 10.18% in the upper layer of the sample to 13.76 in the bottom layer).

#### 4.2.4. Compressive Strength and Modulus of Elasticity

**Figure 16:**
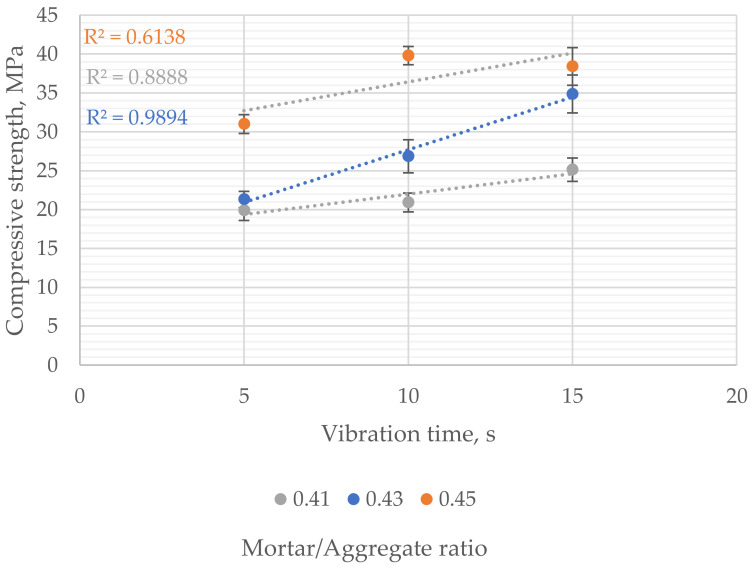
Influence of vibration time on compressive strength of samples with different mortar-to-aggregate ratios.

28-day compressive strength increased with the vibration time, although the significance of changes varied depending on the mortar-to-aggregate ratio. The lowest compressive strength of 19.9–31.0 MPa was observed for samples compacted for 5 s. Samples vibrated for 10 s had compressive strength in the range of 19.6–39.8 MPa and vibrated for 15 s in the range of 23.5–38.4 MPa (Figure 16). The size of the observed changes was strongly correlated with the mortar content in the mix. The strongest effect of increasing vibration time on compressive strength (almost 40% increase) was observed in the mix with a moderate mortar-to-aggregate ratio (0.43).

The compressive strength increased linearly with increased vibration time except for samples with the highest amount of mortar. The nonlinearity of the vibration time’s influence was observed in that case. However, due to the limited number of tested samples and the limitation of the experimental field, a linear function was used to show the trend.

The pervious concrete with the highest amount of mortar and lowest porosity showed the best 28-day compressive strength. In that case, 5 s of vibration was sufficient to obtain a compressive strength of more than 30 MPa. For comparison, samples with the least amount of mortar reached compressive strength ranging from 21.0 MPa to 23.5 MPa. The obtained results showed that the mortar-to-aggregate ratio and the vibration time significantly impact the compressive strength of pervious concrete. However, the effect of mortar content in the mix was significantly stronger than vibration time.

**Figure 17:**
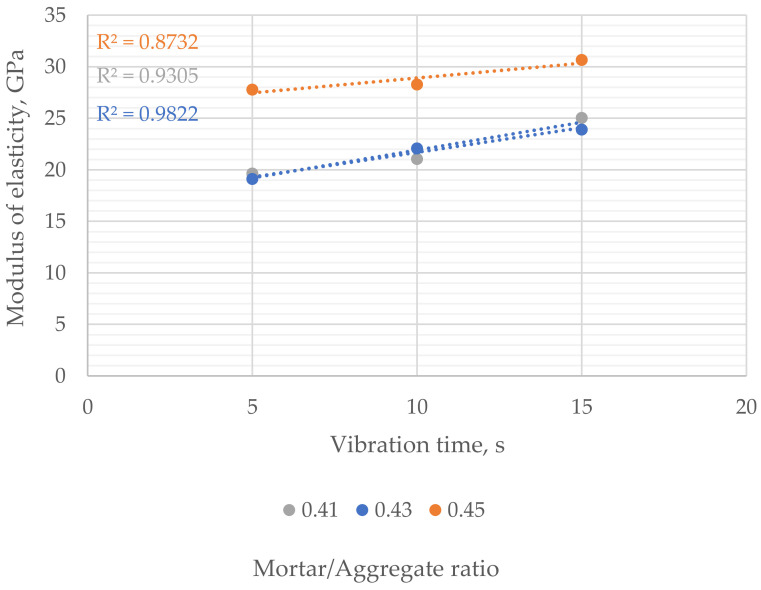
Influence of vibration time on the modulus of elasticity of samples with different mortar-to-aggregate ratios.

The modulus of elasticity ranged from 19.1 to 30.6 GPa—the highest results were obtained for samples with the highest mortar-to-aggregate ratio with the lowest porosity. The results of samples with 0.41 and 0.43 mortar-to-aggregate ratios were similar regardless of the vibration time. The modulus of elasticity increased liner with the vibration time (Figure 17).

#### 4.2.5. Water Permeability

**Figure 18:**
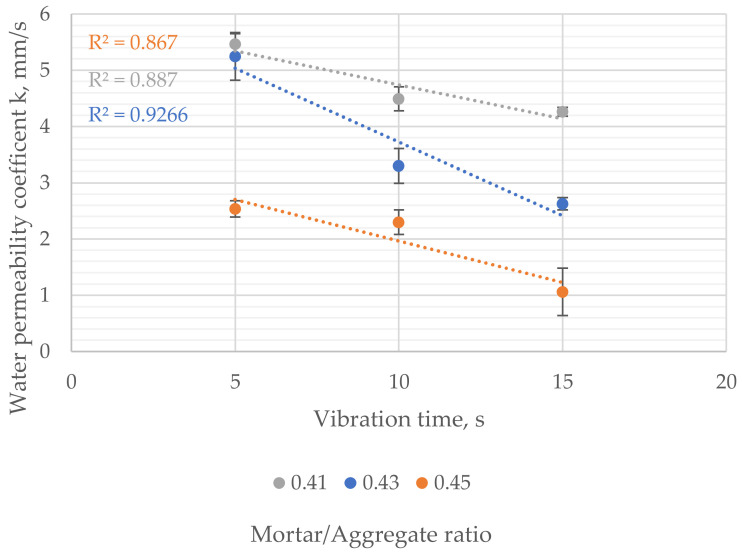
Influence of vibration time on water permeability coefficient of samples with different mortar-to-aggregate ratios.

The water permeability coefficient (k) varied from 1.1 to 5.5 mm/s. It was significantly influenced by vibration time and mortar-to-aggregate ratio (Figure 18). Samples with the mortar-to-aggregate ratio 0.41 were characterized by a 4.3–5.5 mm/s water permeability coefficient. Increasing the mortar content resulted in a k’s factor in the range of 2.6–5.3 mm/s in the case of moderate mortar content and 1.1–2.5 mm/s in the case of samples with the highest amount of mortar. The results suggested a linear correlation between each concrete mix’s vibration time and water permeability coefficient. Water permeability decreased as vibration time increased.

## 5. Discussion

### 5.1. Usefulness of Modified Vebe Method to Determine the Optimal Vibration Time of Pervious Concrete

The authors proposed a modified vebe method to quantify the vibration time needed to overcompact the pervious concrete sample. Knowing such time would help to determine the optimal time of vibration. It could be useful in adjusting the rheological parameters of a fresh mix by, e.g., the addition of admixtures. The method could also be used in everyday quality control of pervious concrete.

The Vebe method was used earlier in the studies [41,42,43] to determine the workability of pervious concrete mixes. Ximenes et al. [41] used a standardized vebe method to characterize the workability of fresh mixes of pervious concrete. Authors reported vebe time ranged from 6.17 to 9.67 s, decreasing with an increase in water to water-to-cement ratio of the mixes. Cheng et al. [42] studied the influence of recycled aggregate substitution on the properties of pervious concrete. Authors reported vebe times from 3.1 to 5.9 s for mixes with aggregate to cement ratio ranging from 3.90 to 4.11, concluding that vebe time for pervious concrete with recycled aggregate was, on average, lower than with natural aggregate.

In the standardized vebe method, [31] tested sample is weighted with a mass of plastic disk and vertical rod, equal to approx. 4 kg, and the time is measured for the transparent disc to be fully covered with cement paste. During preliminary tests, authors observed that by the time cement paste covers the bottom of a plastic disc in the standardized vebe method, the bottom of the sample was often clogged, which was not visible during the test. Hence, to replicate right below the plastic disk compacting conditions that normally occur near the bottom surface of the mold, the authors proposed an additional weight of approx. 8 kg.

The presented method allowed us to quantify the consistency of pervious concrete mixes with mortar-to-aggregate ratio varying from 0.41 to 0.43. Recorded vebe times ranged widely, from 5.1 to 22.1 s, which made it possible to identify even slight differences between rheological parameters of examined mixes with the same mortar content.

**Figure 19:**
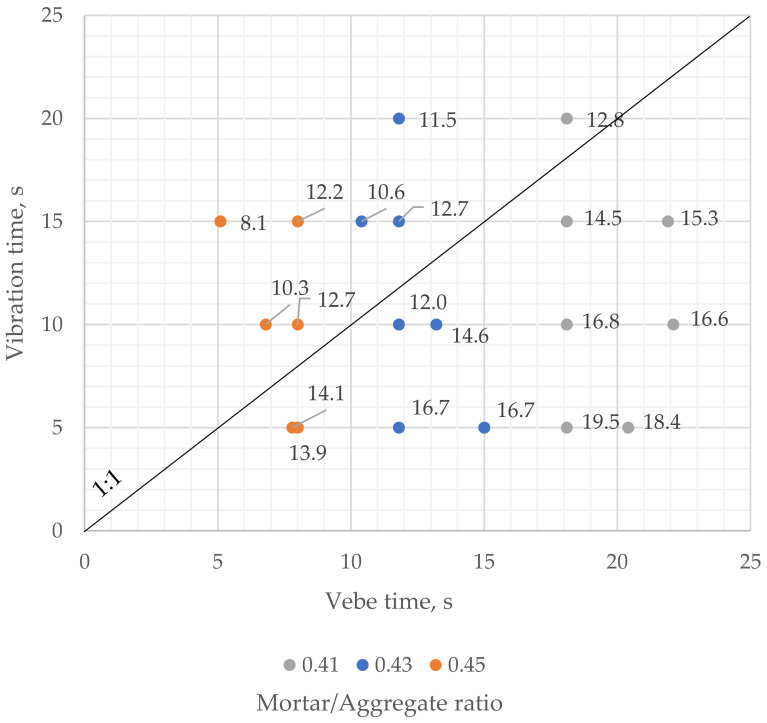
Vibration time vs. vebe time for samples with different mortar to aggregate ratios. Values on the chart indicate the average fresh mix porosity of samples.

The average fresh mix porosity of samples compacted for a longer time than determined by the modified vebe method was lower than 13.0% (Figure 19), which was close to the limit value of 12.0%, indicating clogging effect occurrence in the presented research. However, in one case, of a sample with a 0.43 mortar-to-aggregate ratio, the average porosity of fresh mix was 12.0%, though compaction time was about 18% lesser than vebe time. It suggests that the recommended vibration time should be lesser than the modified vebe time to prevent pervious concrete from clogging.

**Figure 20:**
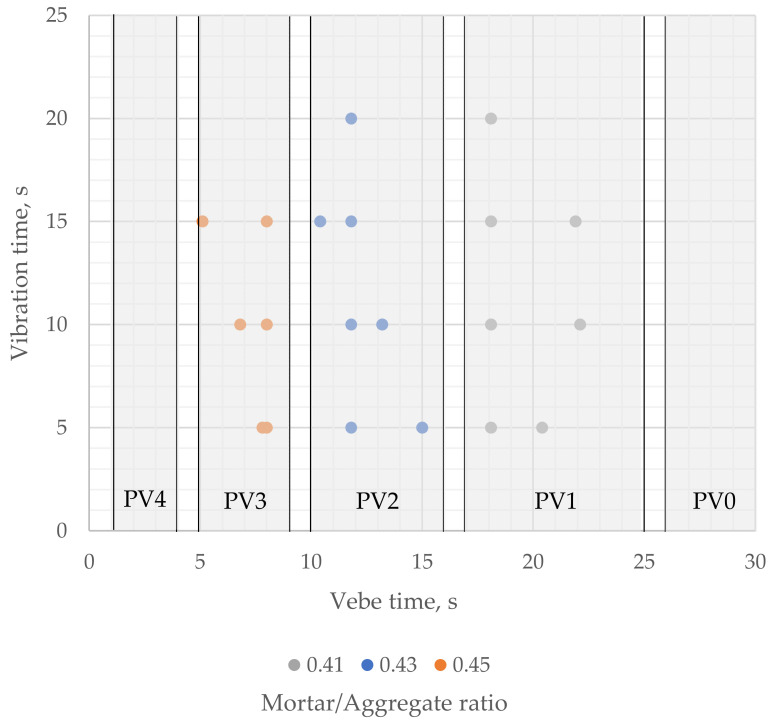
Proposition of modified vebe’s consistency classes of pervious concrete with vibration time vs. vebe time results for samples with different mortar-to-aggregate ratios.

The results indicate that pervious concrete consistency measured by the modified vebe method should be specified in classes rather than target values. Based on the results rounded up to the nearest unity, authors suggest five classes of pervious concrete’s consistency measured by the described method (Figure 20): PV4 (1–4 s), PV3 (5–9 s), PV2 (10–16 s), PV1 (17–25 s), PV0 (>25 s). The suggested consistency classes are justified as analogous to, e.g., slump classes for conventional concrete and could help determine the optimal vibration time. The optimal vibration time should be related to the mix’s consistency rather than specified as a fixed value, as in the earlier studies considering the effect of compaction effort on the properties of pervious concrete [25,27].

**Figure 21:**
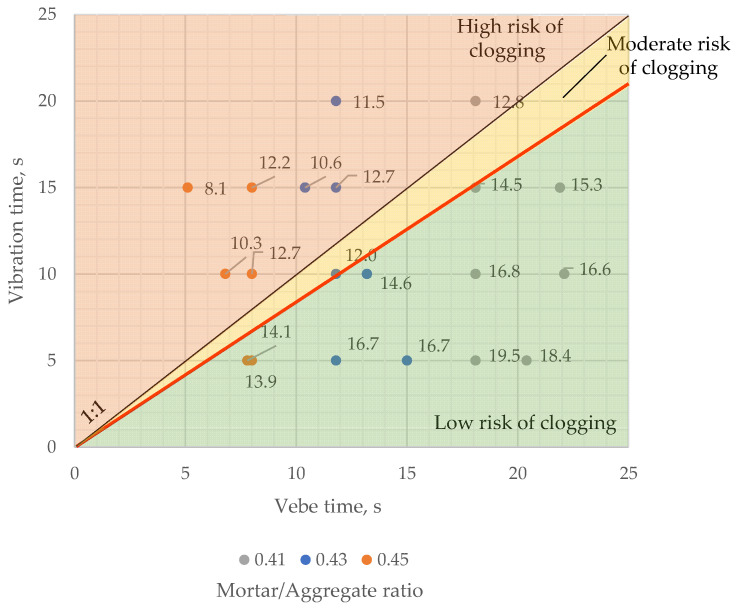
Vibration time vs. vebe time for samples with different mortar to aggregate ratios. Color-coded areas of low (green), moderate (yellow), and high (red) risk of clogging with fixed limit value (red line) of 80% of vebe time.

As mentioned, optimal vibration time should be less than modified vebe time. However, as shown in Figure 21, a fixed limiting value of 80% of vebe time, regardless of the fresh mix consistency, would be rather insufficient. For example, a sample with a 0.41 mortar-to-aggregate ratio and vebe time of approximately 18 s was compacted for 80% of the vebe time, and its average fresh mix porosity was 14.5%. It suggests that the optimal vibration time for mixes with lower fluidity could be closer to the vebe time. On the other hand, 80% of vebe time may cause clogging of mixes with high fluidity.

**Figure 22:**
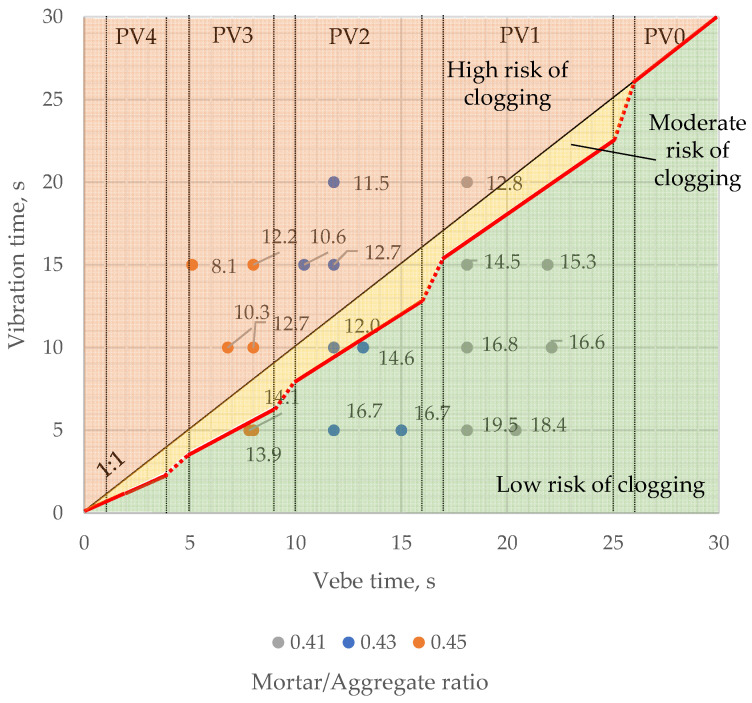
Vibration time vs. vebe time for samples with different mortar to aggregate ratios. Color-coded areas of low (green), moderate (yellow), and high (red) risk of clogging with limit value (red line) depending on consistency class.

Hence, the authors propose to link the optimal range of vibration time with the consistency class of pervious concrete (Figure 22). In that approach, the value limiting the risk of a clogging effect is different for each of the proposed consistency classes—60% of vebe time for PV4 class, 70% for PV3, 80% for PV2, 90% for PV1, and 100% vebe time for PV0 class.

Based on the results, a numerical model was created using a multivariate regression method. As a result of calculations, the regression Equation (6) describing the dependence of the porosity (*P*, %) of the mixture on the vebe time (*VEBT*, s) and the vibration time (*VBT*, s) of the mixture was obtained.

(6) P=0.404·VEBT−0.381·VBT+12,462,

where

P—fresh mix porosity, %

VEBT—Modified vebe time, s

VBT—Vibration time, s

**Figure 23:**
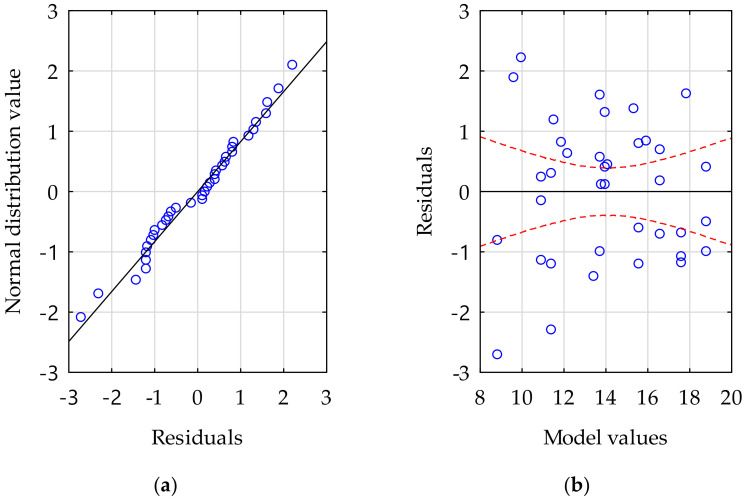
(**a**) normality of residuals (**b**) values of residuals vs. model values from regression Equation (6) charts. Red dotted lines indicate the range of 95% confidence interval. Blue circles indicate experimental points.

**Figure 24:**
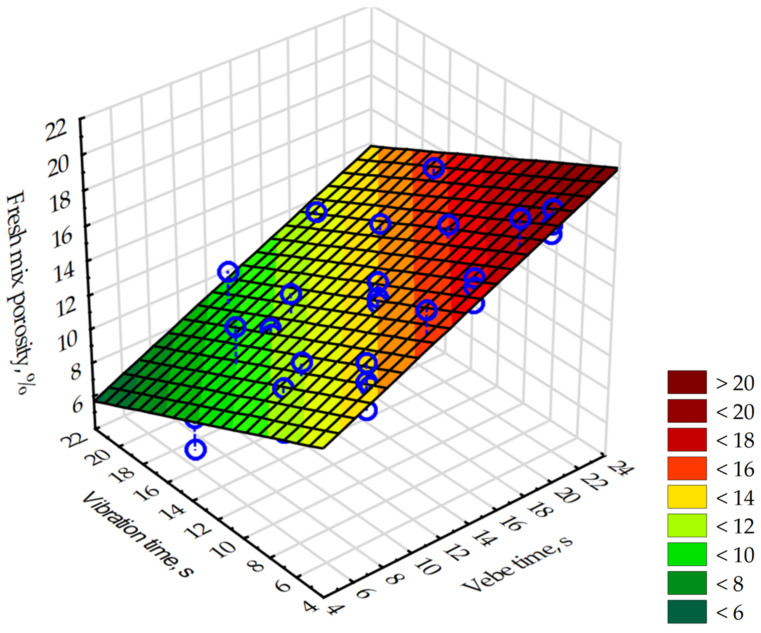
The dependence of the fresh mix porosity on the vibration time and vebe time of pervious concrete, as described by Equation (6). Blue circles indicate experimental points.

The presented model was characterized by a very good fit to the experimental data, which was confirmed with an adjusted coefficient of determination R_a_^2^ of 0.86. The statistical significance of the model was confirmed with *p* << 0.05. The obtained residuals had a normal distribution (Figure 23a) and met the homoskedasticity condition (Figure 23b). The model described by the regression Equation (6) is shown in Figure 24 and could be used for predicting the desirable porosity of fresh mix based on the modified vebe time and vibration time on the vibration table with earlier described parameters.

### 5.2. Shaping the Properties of Pervious Concrete through Its Porosity

Pervious concrete can be characterized by properties unknown in regular concrete technology [44]. With increased porosity, which forms an open network throughout the composite, a dynamic flow of fluids can occur, allowing for pervious concrete’s use as a permeable material for many different applications—as a much bigger area of concrete is exposed to the conditions of external environment than in the case of regular concrete, this type of cementitious material can be used for water management issues [45] as well as for air purification passive systems by inclusion of photocatalytic materials in concrete’s structure [46,47]. As the aforementioned flow occurs, part of the volume of pervious concrete is not filled with aggregate or cement matrix. Due to this, a smaller portion of the material’s volume can carry the load if exposed to it, reducing the material’s overall strength. As there is a need to include a high volume of large pores within the composite, fine and coarse aggregate content in pervious concrete is much lower than in the case of regular concrete. As a result, any mechanical load is transferred through aggregate and a layer of cement paste/mortar covering the grains of coarse aggregate. Its chemical properties, depending on the composition of the binder, and physical—the thickness of the cement paste layer on the coarse aggregate, influence the overall properties of the material, porosity in particular [15,48].

The effective porosity of pervious concrete has the most significant influence on its properties [14]. Its shaping process needs to take into account both the properties of components of the mix–void coefficient of aggregate and its properties [49], rheological properties of cement paste or mortar [50], and compaction method and time, as well as mass ratios between different components of the mix, mainly mortar-to-aggregate ratio and cement-to-sand ratio. All those factors impact the susceptibility of the porosity shaping of concrete mix and, inevitably, the effective porosity of pervious concrete. In performed research, the influence of two independent variables on different properties of pervious concrete was investigated—a material design variable in the form of a mortar-to-aggregate ratio, describing the relative amount of binder in the concrete mix and a technological factor—compaction time. As it was shown, both of those variables influenced several different properties of the previous concrete. However, all the prepared concretes were characterized by the use of the same type of components, and the ratio between them changed in a relatively narrow scope (mortar-to-aggregate ratio changed in the range of 0.41 to 0.45)—the most significant variable influencing properties of pervious concrete was its effective porosity. Although the material composition and technological process (compaction time) significantly affected the acquired effective porosity of hardened pervious concrete, all tested properties depended on the parameters of the pore network in the prepared material.

**Figure 25:**
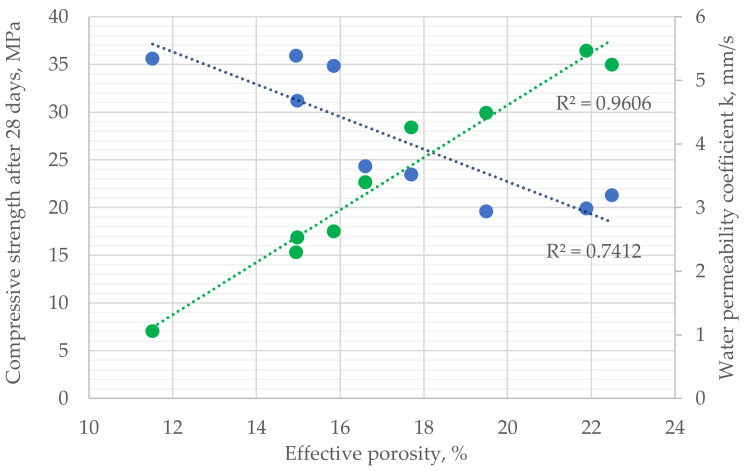
Dependence between the effective porosity of hardened pervious concrete, compressive strength after 28 days, and water permeability coefficient k.

As pervious concrete is mainly used to construct permeable pavements, its water permeability and compressive strength are vital properties to design elements with such functions [13,51]. As shown (Figure 25), both mechanical performance as well as water permeability coefficient k [mm/s] are dependent on the effective porosity of hardened material [24,26]. With an increase in effective porosity of the composite, which can be acquired due to changes in the material composition of the concrete mix—by reducing mortar-to-aggregate ratio—or through a reduction in the compaction time, the compressive strength of the material decreases. In the performed research, the reduction was observed from the value of approx. 35 MPa for effective porosity of approx. 11.5%, to approx. 20 MPa for effective porosity of approx. 22%. This effect takes place due to changes in the macrostructure of the composite. As effective porosity increases, the connection between coarse aggregates made from mortar loses continuity. In the case of mechanical load’s presence, those spots are the first to lose the carrying capacity and contribute significantly to the overall reduction in the strength of the composite. On the other hand, with an increase in the effective porosity, the interconnected pore network increases its volume within the composite, allowing for greater water permeability [19]. In the case of the performed research, the water coefficient of the tested pervious concrete series varied significantly in the range from 1 to 5.5 mm/s, depending on the effective porosity of the concrete.

**Figure 26:**
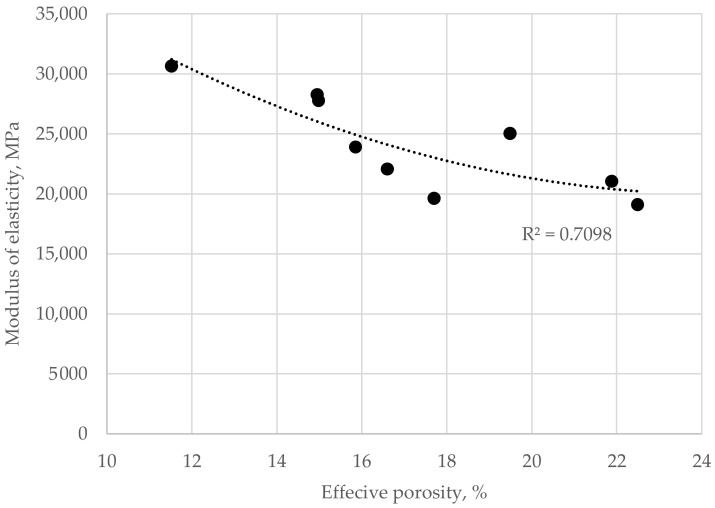
Dependence between the effective porosity of hardened pervious concrete and modulus of elasticity.

The same dependence was true in the case of the modulus of elasticity (Figure 26). With an increase in the effective porosity of pervious concrete, its modulus of elasticity reduces. The same mechanism as in the case of strength performance occurs—all load is transferred through a network of mortar spots connecting coarse aggregate grains, making the performance of a relatively small volume of composite the main constituent of its mechanical performance. Although in the performed research, only one type of binder was used and the range of its content in the composite was relatively narrow, one can imagine that with the change in its properties, both chemical and rheological, the mechanical performance of pervious concrete—even of the same proportions between components of the mix—would differ from obtained results. The reduction in mechanical properties would have the same dependence on the effective porosity of the pervious concrete. However, the range of those changes would be different than in the presented study.

The other aspect worth considering is the influence of effective porosity on the material composition of any given pervious concrete. As the amount of free space results from its designed composition and compaction method, developing the composition of pervious concrete is difficult, especially in the production conditions. Due to high uncertainty regarding the porosity of fresh mix (as it is unknown during the design process), the Authors suggest initially describing concrete’s composition as a set of mass ratios between different components of the concrete mix—depending on the type of pervious concrete by either mortar-to-aggregate ratio and cement-to-sand ratio, or by cement paste-to-aggregate in the case of composition without fine aggregate. The water-to-cement ratio additionally describes concrete. All those proportions accurately describe every pervious concrete mix—its volume depends on its final porosity, properties and amount of components of the mix, and compaction characteristics. Due to this, concrete mix with the same proportion between components and a difference in the porosity can be characterized by a different composition per volume (Table 5).

In the case of performed research, the composition of the concrete mix depended on the proportions between different components of the mix and the porosity of the fresh concrete mix. For concrete mixes of the same ratios between its components, the overall composition of fresh mix per 1 m^3^ changed depending on its porosity, resulting in different masses of, for example, cement in the unit of volume.

## 6. Conclusions

Using pervious concrete as a pavement material positively affects human comfort and water management issues regarding water retention, storage and drainage, and purification. Elements constructed with pervious concrete are also sound absorbers and reduce the heat island phenomenon in urban areas. However, to properly design properties of concrete of that type, its composition and preparation method must be appropriately selected to reach its designed porosity. Based on the conducted research, several conclusions can be made:

- Properties of pervious concrete, mainly compressive strength, permeability coefficient, and modulus of elasticity, depend on the effective porosity of pervious concrete;
- The composition of the pervious concrete mix depends on the proportions between its different components and porosity—concrete of the same ratios between different components can be characterized by different overall composition due to differences in the porosity of fresh mix;
- The effective porosity of pervious concrete and the porosity of fresh concrete mix depends on the material composition of pervious concrete (mortar-to-aggregate ratio) and compaction time;
- The optimal compaction time to acquire designed effective porosity changes along with the material composition of pervious concrete;
- An increase in the content of mortar in the composition of pervious concrete results in a shortening of compaction time required to reach the designed effective porosity;
- The modified vebe method proposed by authors allowed to quantify the consistency of pervious concrete mixes with mortar-to-aggregate ratio varying from 0.41 to 0.43. The proposed method made it possible to identify even slight differences between the rheological parameters of examined mixes with the same mortar content;
- Authors suggest five classes of pervious concrete’s consistency measured by the modified vebe method: PV4 (1–4 s), PV3 (5–9 s), PV2 (10–16 s), PV1 (17–25 s), PV0 (>25 s). The suggested consistency classes are justified as analogous to, e.g., slump classes for conventional concrete and could help determine the optimal vibration time;
- A numerical model proposed by authors allows us to predict the desirable porosity of fresh mix based on the modified vebe time and vibration time on the vibration table.

## Figures and Tables

**Table 1:** Chemical composition of cement used in the research.

Oxide/Compound	wt. %
MgO	1.88
Al_2_O_3_	3.23
SiO_2_	18.99
P_2_O_5_	0.26
SO_3_	3.77
K_2_O	0.89
CaO	63.34
TiO_2_	0.28
MnO	0.08
Fe_2_O_3_	2.83
CuO	0.02
ZnO	0.03
SrO	0.07
LOI	0.01

**Table 2:** Mixtures characterization.

Property/ Composition	M1	M2	M3
Mortar/ Aggregate, -	0.41	0.43	0.45
Cement Paste/ Aggregate	0.28	0.29	0.30
Water/ Cement, -	0.30		
Cement/ Fine aggregate -	1.618		
Superplasticizer content, %mass of cement	0.58		
Fine aggregate content, %mass of aggregate	13.3	13.8	14.4

**Table 3:** Average fresh mix porosity and effective porosity of hardened pervious concrete 100 × 100 × 100 mm samples (Abbreviations: CV—coefficient of variation).

Mortar-to-Aggregate Ratio -	Vibration Time s	Fresh Mix Porosity %	CV %	Effective Porosity %	CV %
0.41	5	18.44	3.23	21.88	2.24
	10	16.62	1.14	19.49	4.59
	15	15.26	5.20	17.70	7.20
0.43	5	16.68	3.41	22.49	3.78
	10	14.59	3.42	16.60	7.35
	15	10.57	5.62	15.85	2.08
0.45	5	13.27	10.64	14.98	13.00
	10	11.01	17.88	14.95	9.85
	15	10.35	32.32	11.84	7.45

**Table 4:** Effective porosity distribution with height (U—upper part, M—middle part, B—bottom part).

Vibration Time s	Sample	Mortar-to-Aggregate Ratio -					
		0.41		0.43		0.45	
		Effective Porosity %					
5	U	24.47	Average	20.66	Average	15.61	Average
	M	24.81	26.48	23.88	23.43	20.63	19.39
	B	30.17		25.74		21.94	
10	U	20.43	Average	17.65	Average	11.99	Average
	M	24.85	23.65	21.20	20.17	16.07	16.21
	B	25.66		21.67		20.56	
15	U	17.98	Average	14.66	Average	10.18	Average
	M	21.65	20.29	14.66	16.19	11.22	11.72
	B	21.24		19.24		13.76	

**Table 5:** Composition of concrete mixes per 1 m^3,^ including average porosity of fresh concrete mix; different shades of gray indicate concrete mixes of the same ratios between components of the mix.

Compact Time	Water-to-Cement	Cement-to-Sand	Mortar-to-Aggregate	Average Fresh Mix Porosity	Cement	Water	Fine Aggregate 0/2	Coarse Aggregate 5/8	SP
**s**	**−**			**%**	**kg/m^3^**				
5	0.3	1.618	0.41	18.44	372	112	230	1504	2.2
10				16.62	380	114	235	1538	2.2
15				15.26	386	116	239	1563	2.2
5			0.43	16.68	380	114	235	1463	2.2
10				14.59	389	117	241	1500	2.3
15				10.57	408	122	252	1571	2.4
5			0.45	13.27	395	119	244	1454	2.3
10				11.01	406	122	251	1492	2.4
15				10.35	409	123	253	1503	2.4

## Data Availability

Available on request.
